# Effect of Drying Operating Conditions on Canola Oil Tocopherol Content

**DOI:** 10.3390/antiox3020190

**Published:** 2014-03-27

**Authors:** Daniela Laoretani, María Fernández, Guillermo Crapiste, Susana Nolasco

**Affiliations:** 1 TECSE—Facultad de Ingeniería—UNCPBA, Av. Del Valle 5737, Olavarría, Buenos Aires 7400, Argentina; E-Mail: danielalaoretani@hotmail.com; 2 CIFICEN (UNCPBA-CONICET), Pinto 399, Tandil, Buenos Aires 7000, Argentina; 3 PLAPIQUI (UNS-CONICET), Camino La Carrindanga km 7, CC 717, Bahía Blanca, Buenos Aires 8000, Argentina; E-Mail: rector@uns.edu.ar

**Keywords:** canola, tocopherol, acidity value, peroxide index, fatty acid composition, drying operating conditions

## Abstract

The aim of this work was to evaluate two operating parameters of seed drying (temperature and initial moisture content) on the tocopherol content of canola oil. The raw material was characterized by moisture, oil, protein, crude fiber and ash content. Seeds at 13.6% and 22.7% moisture content (dry basis, db) were dried at temperatures in the range of 35–100 °C to a safe storage moisture of 7% db. Oil was extracted from each treated sample. The oil extracted from the samples dried at the extreme temperatures was analyzed by means of the acidity value, peroxide index and fatty acid composition, finding no significant differences among treatments or among untreated and treated samples. Tocopherol contents in the oils obtained for all the assayed temperatures were determined. Differences were found for the samples with 22.7% (db) initial moisture content. Except at 35 °C, temperature affected negatively the oil tocopherol content. However, when 13.6% (db) moisture seeds were processed, no significant differences were observed in the amount of this minor oil component among assays.

## 1. Introduction

Canola oil is one of the most important vegetable oils in the global market of edible fats because of its qualities for human consumption. It is characterized by being very low in saturated fat and high in monounsaturated fat, and by containing significant amounts of omega-3 (alpha-linoleic acid) and omega-6 acids (linoleic acid), both essential in the human diet. In addition, canola oil contains high amounts of bioactive compounds, such as polyphenols, phytosterols, tocopherols and other antioxidants [1]. Various studies have shown that free radicals present in the human organism cause oxidative damage to different molecules, such as lipids, proteins and nucleic acids, and thus are involved in the initiation phase of some degenerative diseases. Thus, antioxidant compounds play an important role in the prevention and treatment of some chronic diseases, for example heart diseases, neurodegenerative diseases, aging, cancer and rheumatoid arthritis [1,2,3]. The antioxidant activity of tocopherols in particular is mainly due to their ability to donate their phenolic hydrogen to lipid free-radicals. Unsaturated fatty acid autoxidation reactions generally can be divided into three major reactions: initiation, propagation and termination. The first step (initiation reaction) involves the generation of a radical from an unsaturated fatty acid. The alkyl radicals formed in this stage are very reactive, and they generally combine with available oxygen-giving peroxy radicals. A probable explanation for the mechanism of antioxidant action of tocopherols was proposed [4]. The tocopherol molecule cedes a proton to the peroxy radical, forming the chromanoxyl radical. The chromanoxyl radical may undergo radical-radical coupling with other radicals forming adducts. Thus, each tocopherol molecule can neutralize two peroxy radicals by reactions.

Once the canola is harvested, it is necessary to reduce its moisture content for a safe storage, to between 5.5% and 8.5% wet basis (wb) depending on the storage temperature and the oil content of the seeds [5]. The drying of rapeseeds to obtain improved mechanical characteristics prior to press extraction was studied by Fornal *et al.* [6]. The literature shows divergent data about the effect of the process temperature on the oil quality, measured in terms of its acidity value, peroxide index, and tocopherol content, of different seeds due to the interaction between variables such as dryer characteristics, temperature and required time to reach a particular moisture content, among others [5,6,7,8,9,10,11,12,13]. While it has been found that temperature and storage time generated a decrease in tocopherol content in the oil of wheat germen samples at 27 °C and 45 °C [12], treatments like the toasting of soybean seeds between 100 and 140 °C generated an increase in tocopherol content [13].

The aim of this work was to analyze the influence of two operating variables of the drying process (temperature and moisture) on the tocopherol content of canola oil.

## 2. Experimental Section

### 2.1. Sample Characterization

Winter canola seeds (Barrel variety) from a 10 kg bag were characterized. The initial moisture and oil content of the ground seeds (0.420–1.00 mm) were determined by IUPAC 1.121 and 1.122 standard methods [14], respectively. Standard AOCS official methods were used to determine crude fiber, protein (nitrogen × 5.3 factor) and ash contents (AOCS Ai 4-91, AOCS Ba 6-84 and AOCS Ba 5a-49 [15], respectively).

### 2.2. Oil Characterization

#### 2.2.1. Fatty Acid Composition

Fatty acid composition was determined by gas chromatography and expressed as a percentage of total fatty acid content [16].

#### 2.2.2. Physico-Chemical Parameters

The acidity value and peroxide index were determined following the methods described in IUPAC 2.201 [14] and AOCS Cd 8 [15], respectively.

#### 2.2.3. Tocopherol Concentration

The tocopherol concentration in canola oil was determined by normal-phase high performance liquid chromatography (HPLC), following AOCS Ce 8-89 standard method [15]. A Hewlett Packard Series 1050 equipment with fluorescence detector was used (excitation wavelength: 290 nm, emission wavelength: 300 nm), with a HiCHROM column, Lichrosorb Si 60, 250 × 4.6 mm i.d. and 5 μm particle size. Hexane:isopropanol (99.5:0.5 v/v) was used as mobile phase, with a flow rate of 1.5 mL/min. The identification of the peaks in the chromatogram was carried out considering the retention times and/or patterns of the different tocopherol isomers. The quantification of the tocopherol isomers was performed by the external standard method using α-tocopherol as reference (Sigma T3251, 95% purity; AOCS Standard Method Ce 8-89 [15]).

### 2.3. Drying Assays

**Figure 1:**
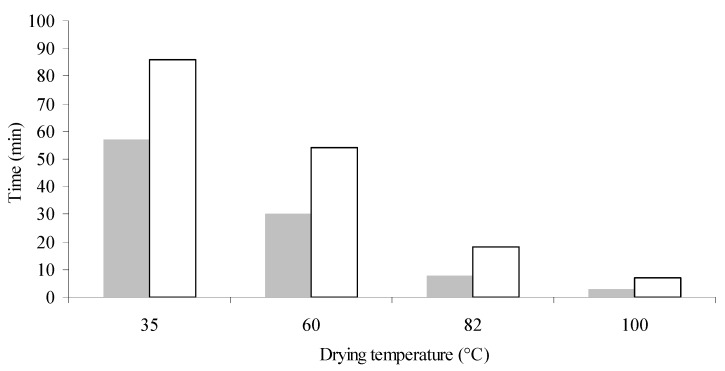
Drying times of canola samples at an initial moisture level of 13.6% db (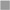) and 22.7% db (□) and final moisture content of 7% db. Eight independent drying experiments. *n* (number of replicates of each measurement): 2.

Water was added to the seeds in order to reach a moisture level of 13.6% and 22.7% (db). Then the seeds were stored for 72 h in closed containers at 8 °C to homogenize the moisture in the samples. Afterwards, the samples were dried to a safe storage moisture level of 7% db [5] in a forced convection air oven at different temperatures (35, 60, 82 and 100 °C). Time needed for each experimental condition is shown in Figure 1. Oil was extracted from each sample by Soxhlet using hexane as solvent (IUPAC 1.122 [14]), the miscella was collected and the solvent was evaporated in an R-3000 Büchi vacuum rotary evaporator (Switzerland), not exceeding 50 °C. Residual hexane was removed in a nitrogen stream to constant weight. The amount of oil extracted was measured gravimetrically using a Sartorius balance (PB211D model, precision: 0.1 mg). The oil extracted was characterized by physico-chemical parameters, such as acidity value and peroxide index, fatty acid composition (gas chromatography) and tocopherol content (HPLC), as described above.

### 2.4. Statistical Analysis

Values represent the means and standard deviations of two replicates. The statistical analysis was carried out by analysis of variance using the Infostat software [17]. Tukey method was used to compare the means of pairs of treatments with a significance level of *p* < 0.05.

## 3. Results and Discussion

### 3.1. Sample Characterization

The canola seed characterization is shown in Table 1, with all the parameters expressed on a dry basis (db). The moisture content of the seeds at harvest was in the 8.0%–8.2% db range. The oil content was 45.2% db, which is in the expected range for rapeseeds (40%–60% db [18]). The protein content was 18.7% db, corresponding to a value of 30.2% in defatted wet basis, lower than the values of 38%–43% reported by Thakor *et al.* and closer to the lower limit of 30.5%–40.5% reported by Daun *et al.* for fall-harvested canola [19,20].

**Table 1:** Characterization of canola seeds (Barrel variety).

Determination (% db)	Canola
Moisture	8.1 ± 0.1
Oil	45.2 ± 0.9
Protein	18.7 ± 0.6
Crude fiber	20.3 ± 0.5
Ash	3.7 ± 0.1

### 3.2. Drying Assays

#### 3.2.1. General Oil Characterization

Experimental data of fatty acid composition, acidity value and peroxide value of the oil extracted from the pretreated seed samples for the extreme temperatures tested during the drying process are shown in Table 2. Letters of comparison obtained by Tukey’s test were not indicated in the table because there were no differences within each column, so they were all the same indicating no significant differences among assays for each determination. For example, the untreated sample presented the following fatty acid composition: 4.25% ± 0.09% palmitic acid (C16:0), 1.80% ± 0.06% stearic acid (C18:0), 72.21% ± 0.13% oleic acid (C18:1), 15.17% ± 0.03% linoleic acid (C18:2) and 6.55% ± 0.09% linolenic acid (C18:3), an acidity value of 0.49 ± 0.02 mg KOH/g oil, and no peroxide value was detected. These values were not significantly affected by the drying pretreatment under the operating conditions used in the present work (*p* > 0.05). These results are similar to those reported by Sutherland and Ghaly, who dried rapeseed and sunflower seeds up to 80 °C [10]. Pathak *et al.* did not find differences in free fatty acid content when rapeseed was dried from 20% to 8% of initial moisture at the 50–95 °C range, but they did observe differences in the acidity content of the oil from treated samples compared to the control sample [8].

**Table 2:** Characteristics of canola oil extracted after the drying pretreatment of seeds at different initial moisture contents (W) and drying temperatures (T) and final moisture content of 7% db.

W	T	C16:0	C18:0	C18:1	C18:2	C18:3	AV	PV
(% db)	(°C)	(%)	(%)	(%)	(%)	(%)		
13.6	35	4.13	1.74	72.35	15.14	6.64	0.50	*ND*
13.6	100	4.25	1.86	72.26	15.16	6.47	0.51	*ND*
22.7	35	4.27	1.86	72.11	15.15	6.61	0.49	*ND*
22.7	100	4.33	1.73	72.13	15.19	6.63	0.51	*ND*

W, initial moisture content expressed in % dry basis (db); T, drying temperature in °C; C16:0, palmitic acid; C18:0, stearic acid; C18:1, oleic acid; C18:2, linoleic acid; C18:3, linolenic acid; AV, acidity value in mg KOH/g oil; PV, peroxide value in meq O_2_/kg oil. *ND*: not detected. 4 independent drying experiments. *n* (number of replicates of each measurement): 2.

#### 3.2.2. Tocopherols

Total tocopherol content was 700 ± 3 μg/g oil and 699 ± 3 for untreated samples of 13.6% and 22.7% (db) moisture content, respectively. Only α- and γ-tocopherols were found, γ-tocopherol being the most abundant (57%–59% of total tocopherols).

**Figure 2:**
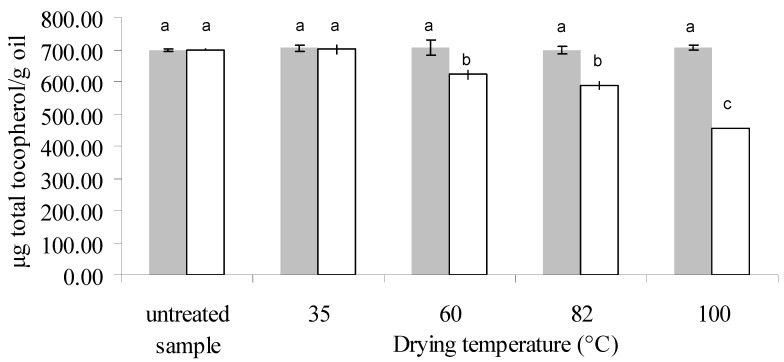
Total tocopherol concentration in the oil extracted from untreated and treated samples of canola, at an initial moisture level of 13.6% db (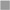) and 22.7% db (□) and final moisture content of 7% db in all treated samples. Different letters indicate significant differences (Tukey’s test, *p* < 0.05). Eight independent drying experiments. n (number of replicates of each measurement): 2.

**Figure 3:**
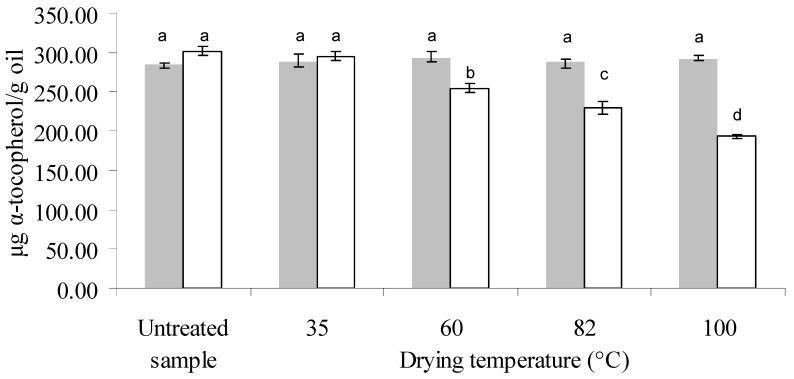
Alpha-tocopherol concentration in the oil extracted from untreated and treated samples of canola, at an initial moisture level of 13.6% db (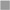) and 22.7% db (□) and final moisture content of 7% db in all treated samples. Different letters indicate significant differences (Tukey’s test, *p* < 0.05). Eight independent drying experiments. *n* (number of replicates of each measurement): 2.

**Figure 4:**
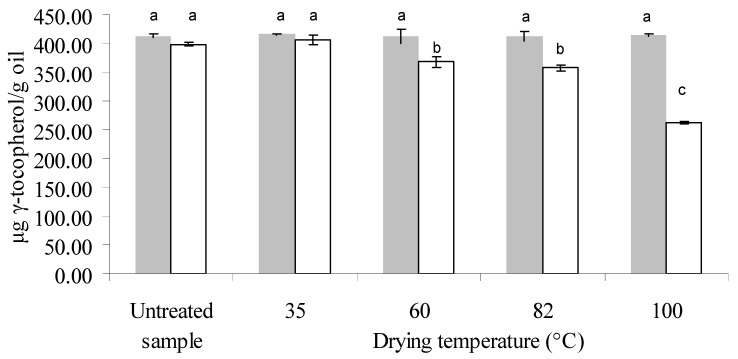
Gamma-tocopherol concentration in the oil extracted from untreated and treated samples of canola, at an initial moisture level of 13.6% db (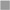) and 22.7% db (□) and final moisture content of 7% db in all treated samples. Different letters indicate significant differences (Tukey’s test, *p* < 0.05). Eight independent drying experiments. *n* (number of replicates of each measurement): 2.

Figure 2, Figure 3, Figure 4 show the amount of these components in the oil from untreated and dried samples. By means of ANOVA, it was found that the initial moisture content, drying temperature and their interaction were significant on the total, α- and γ-tocopherol content of the oil (*p* < 0.05). In the samples with lower moisture (13.6% db), no significant differences were observed among the different treatments or with the untreated sample, both for total tocopherols and for each isomer present.

On the other hand, a significant variation in total, α- and γ-tocopherol content as a function of drying temperature was found for the samples with an initial moisture level of 22.7% db (Figure 2, Figure 3, Figure 4 respectively). Gamma-tocopherol followed the same pattern as total tocopherol content (same letters indicate no significant differences) due to its abundance in this oil (57%–59%), whereas α-tocopherol exhibited a greater effect of temperature: for example at 82 °C (temperature at which the pattern changes) it presented a decrease of 24%, and γ-tocopherol showed a decrease of 10%, resulting in a 16% decrease in total tocopherols.

This difference in temperature effect between samples at different initial moisture contents (IM) could be due to the time the seed was exposed to the drying conditions. For example, at 100 °C the drying process lasted 7 min when IM was 22.7% (db), whereas samples at 13.6% db (IM) could reach the desirable humidity in 3 min (Figure 1).

For the sample with higher IM (22.7%, db), although it was exposed for the longest time when the lower temperature was used (86 min at 35 °C), these drying conditions did not produce a significant difference in tocopherol content between the untreated and treated samples, whereas temperatures from 60 °C to 100 °C had a negative influence in this respect both on total tocopherol and each isomer. Several authors reported a degradation effect of tocopherols due to temperature. Roasting of gourd seeds at high temperature (140 °C) for 5 min produced a loss of tocopherol content of 41% and 36% for α- and γ-tocopherol, respectively, close to the loss percentage found in this work at 100 °C for 7 min (36% and 34% for α- and γ-tocopherol, respectively) [21]. Capitani *et al.* found a negative effect of temperature on the tocopherol content of wheat germ oil during storage of wheat germ (13.7% db IM) at a temperature range of 27–45 °C for longer periods of time than those used in this work (7 to 35 days) [12]. However, these results differ from those reported by Prior *et al.*, who observed a relatively uniform tocopherol content at temperatures between 80 and 100 °C during pretreatments (heating and preheating/flaking/cooking and barrel temperature between 80 and 127 °C during canola pressing [22]). However, they studied the treatment for a longer heating time (30 min), and they used closed containers. On the other hand, Miranda *et al.* observed an increase in tocopherol content with drying temperature in quinoa seeds within a similar temperature range (40 to 80 °C), and this increase was more pronounced above 60 °C [23]. This could be attributed to the fact that the matrix of canola seeds is very different from that of quinoa, which contains 8% oil and a vitamin E content of 5 mg/100 g dry matter. Likewise, when Chu applied a toasting pretreatment to soybeans at a higher temperature range (100–140 °C), they observed an increase in tocopherol content with toasting temperature [13].

These results indicate that the effect of the conditioning temperature on tocopherol content depends on the type of seed being treated, initial moisture level, time of treatment and type of processing. Depending on the matrix of the material under study, a significant amount of vitamin E linked to proteins or phospholipids could be released by the heat treatment [23], or tocopherols could show degradation due to temperature [21,24], making it necessary to study the different processing conditions for each species.

## 4. Conclusions

The forced-convection air drying of canola seeds at different temperatures between 35 and 100 °C did not affect the oxidative parameters of the oil (acidity value and peroxide index) or the fatty acid composition of the oil at the initial moisture levels studied (13.6% and 22.7% db).

At high initial moisture (22.7% db), tocopherol content was affected negatively by the drying temperature in the 35–100 °C range, except at the lowest temperature studied, indicating that the best condition for this pretreatment is 35 °C in order not to affect the amount of tocopherol in the crude oil when the moisture content of the sample is high. At lower moisture levels, it is possible to apply a drying treatment at 100 °C for short periods of time (3 min) without significantly affecting the tocopherol content.

As a perspective, it would be relevant to study the effect of this process on other important antioxidants present in canola oil, such as phytosterols or polyphenols.
